# Deciphering the intersystem crossing in near-infrared BODIPY photosensitizers for highly efficient photodynamic therapy†
†Electronic supplementary information (ESI) available: Experimental details of synthesis, characterization and supplementary figures. See DOI: 10.1039/c8sc04840a


**DOI:** 10.1039/c8sc04840a

**Published:** 2019-01-22

**Authors:** Xiaofei Miao, Wenbo Hu, Tingchao He, Haojie Tao, Qi Wang, Runfeng Chen, Lu Jin, Hui Zhao, Xiaomei Lu, Quli Fan, Wei Huang

**Affiliations:** a Key Laboratory for Organic Electronics and Information Displays , Institute of Advanced Materials (IAM) , Jiangsu National Synergetic Innovation Center for Advanced Materials (SICAM) , Nanjing University of Posts & Telecommunications , Nanjing 210023 , China . Email: iamqlfan@njupt.edu.cn; b Key Laboratory of Flexible Electronics (KLOFE) , Institute of Advanced Materials (IAM) , Jiangsu National Synergetic Innovation Center for Advanced Materials (SICAM) , Nanjing Tech University (NanjingTech) , Nanjing 211816 , China; c College of Physics and Energy , Shenzhen University , Shenzhen 518060 , China; d Shaanxi Institute of Flexible Electronics (SIFE) , Northwestern Polytechnical University (NPU) , 127 West Youyi Road , Xi'an 710072 , China

## Abstract

Deciphering singlet-to-triplet intersystem crossing (ISC) in organic near-infrared photosensitizers (PSs) is of fundamental importance in the designing of high-performance PSs to boost the clinical usage of photodynamic therapy (PDT). However, in-depth investigations of the ISC dynamics in near-infrared PSs have not been performed to date. Here, systematical investigations of the ISC dynamics in organic near-infrared BODIPY derivatives are presented, in which a multi-channel yet remarkably efficient ISC process is revealed by ultrafast femtosecond transient absorption (fs-TA) spectroscopy and theoretical calculation. The fs-TA verifies an exceptionally enhanced ISC efficiency (*Φ*_ISC_ = 91%) in iodine-substituted BODIPY (**2I-BDP**) which is further supported by the calculation results. This endows **2I-BDP** with an ultrahigh singlet oxygen quantum yield (*Φ*_Δ_ = 88%), thus enabling a proof-of-concept application of highly efficient PDT *in vivo* under ultralow near-infrared light power density (10 mW cm^–2^). The in-depth understanding of ISC dynamics in organic near-infrared materials may provide valuable guidance in the designing of novel organic theranostic materials for clinical cancer treatment.

## Abstract 2

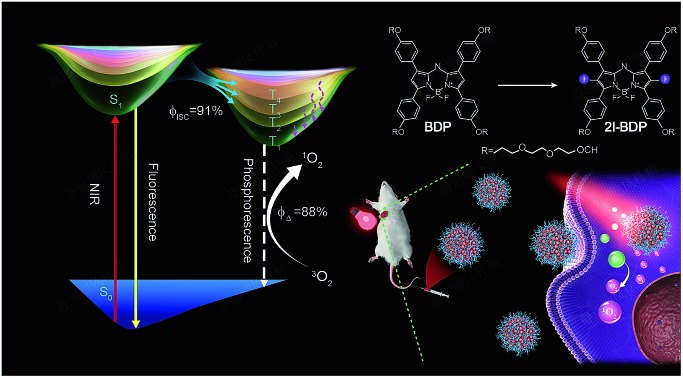
A multi-channel yet exceptionally efficient intersystem crossing process was experimentally and theoretically revealed in near-infrared BODIPY photosensitizers for highly efficient photodynamic therapy.

## Introduction

Photodynamic therapy (PDT) is a rapidly developed, clinically approved therapeutic modality which utilizes the photoexcitation of an external photosensitizer (PS) by appropriate light to produce a highly localized cytotoxicity toward malignant cells.^1^–^3^ Typically, the PDT efficiency highly relies on the singlet oxygen (^1^O_2_) quantum yield (*Φ*_Δ_) of the PS.^4^ From a practical application point of view, an ideal PS should simultaneously possess strong near-infrared (NIR, 650–950 nm) absorption and high *Φ*_Δ_ because NIR light has much deeper tissue penetration than visible light.^5^,^6^ However, most of the available near-infrared PSs always have low *Φ*_Δ_ due to the trade-off between the NIR-absorption and *Φ*_Δ_, and usually require a relatively high power density (>100 mW cm^–2^) and thus greatly impede the practical application of PDT.^7^,^8^ Therefore, simultaneously intensifying the NIR absorption and amplifying the *Ф*_Δ_ of near-infrared PSs is urgently desirable to boost the practical application of PDT.

To date, a primary challenge to achieving high *Φ*_Δ_ in near-infrared PSs lies in how to maximize their singlet-to-triplet intersystem crossing (ISC) quantum yield (*Φ*_ISC_), because it is an energy transfer between the excited triplet sensitizer and ground-state molecular oxygen (^3^O_2_) to yield ^1^O_2_.^9^,^10^ Therefore, it is of fundamental importance to decipher the underlying ISC dynamics in near-infrared PSs for pursuing higher *Φ*_Δ_. However, in-depth investigations of the ISC dynamics in near-infrared PSs have not been performed to date, although incorporating heavy metals (*e.g.*, Ru or Ir) or special organic moieties (*e.g.*, aromatic aldehydes and halogens) into chromophores was used to improve *Φ*_Δ_.^11^ In addition, the as-prepared metal-containing complexes generally possess inherent drawbacks of extremely high cost and unknown heavy metal-induced toxicity concerns,^12^ which is obviously unfavorable for clinical usage of PDT application. In view of these drawbacks, despite the unsatisfactory *Φ*_Δ_,^13^ recently organic NIR-absorptive materials have become an attractive candidate as PSs for PDT owing to their good biocompatibility, biodegradability, and structural flexibility.^7^,^8^,^14^,^15^ Given that, it will become necessary to decipher the underlying ISC dynamics in organic near-infrared materials to boost the development and clinical usage of PDT.

**Scheme 1:**
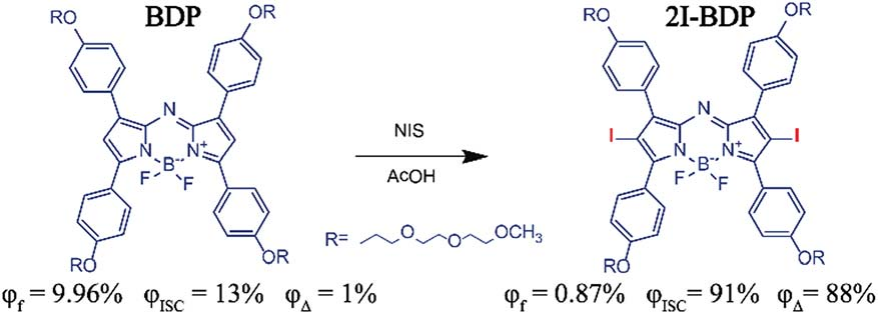
Molecular structures and photophysical properties of **BDP** and **2I-BDP**.

Aza-boron-dipyrromethene (aza-BODIPY) derivatives as popular NIR-absorptive materials have attracted ever-growing interest for PDT,^15^ because *meso*-nitrogen atoms in aza-BODIPY could prolong its absorption and emission peak into NIR. Here, we experimentally and theoretically demonstrated multi-channel yet remarkably efficient ISC dynamics in organic near-infrared BODIPY derivatives for highly efficient *in vivo* PDT. We firstly designed and synthesized organic aza-BODIPY (**BDP**) and iodine-substituted one (**2I-BDP**), which both possess a very strong near-infrared light absorption band ranging from 650 to 720 nm and relatively weak fluorescence around 730 nm. As compared with the parent near-infrared **BDP**, **2I-BDP** exhibited an outstanding ISC feature but relatively weak fluorescence around 720 nm owing to the iodine-induced heavy atom effect which facilitated ISC and then quenched fluorescence. Ultrafast femtosecond transient absorption (fs-TA) spectroscopy, in cooperation with theoretical calculation, revealed a highly efficient *Φ*_ISC_ (91%) of **2I-BDP**. Such a remarkably enhanced *Φ*_ISC_ endows **2I-BDP** with an ultrahigh singlet oxygen quantum yield (*Φ*_Δ_ = 88%), thus enabling a proof-of-concept application of highly efficient PDT *in vivo* under ultralow near-infrared light power density (<10 mW cm^–2^) (Scheme 1).

## Results and discussion

### Characterization of **2I-BDP**

BODIPY derivatives have drawn enormous attention in biomedical applications owing to their attractive features including facile synthesis, structural versatility and excellent spectroscopic properties.^16^ In this study, we replaced the *meso*-carbon atom with the *meso*-nitrogen atom in an attempt to prolong its absorption maximum into NIR (Fig. S1†).^17^ Meanwhile, an alkoxy side-chain was added into the BODIPY conjugated backbone to improve its water-solubility and biocompatibility. The synthesis and characterization are demonstrated in Fig. S2–S7.†

**Fig. 1:**
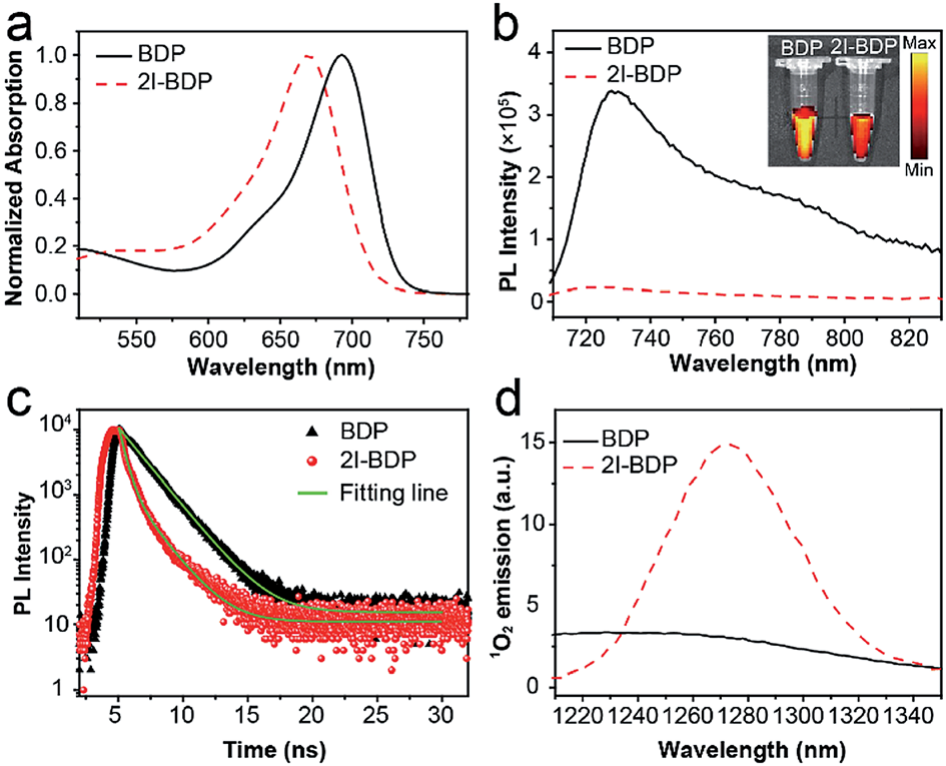
(a) Normalized absorption spectra of **BDP** and **2I-BDP** in methanol. (b) Photoluminescence (PL) spectra of **BDP** and **2I-BDP** in methanol. Inset is their digital photos irradiated by corresponding absorption maxima. (c) Time-resolved photoluminescence decay transients of **BDP** and **2I-BDP** in the specific emission ranges. The optical excitation is performed with 650 nm pump pulses (50 fs, 50 MHz). (d) Characteristic ^1^O_2_ emission of **BDP** and **2I-BDP** in the deuterated method.

The absorption spectra of **BDP** and **2I-BDP** are presented in Fig. 1a with their absorption maxima at 690 and 670 nm, respectively. As shown in Fig. S8,† **2I-BDP** exhibited more intense NIR absorption (*ε* = 8.1 × 10^4^ M^–1^ cm^–1^ at 670 nm) with respect to the parent **BDP** (*ε* = 3.9 × 10^4^ M^–1^ cm^–1^ at 690 nm). The photoluminescence (PL) spectrum in Fig. 1b showed the maximum fluorescence emission at 730 nm for **BDP** and 720 nm for **2I-BDP**. The inset digital photos in Fig. 1b visually revealed a stronger fluorescence in **BDP** as compared with **2I-BDP**. The final absolute PL quantum yield of **BDP** and **2I-BDP** was determined to be 9.96% and 0.87%, respectively. We ascribed this 10-fold decreased PL quantum yield (9.96% *vs.* 0.87%) to the heavy-atom effect which depopulated the singlet excited states in **2I-BDP** through nonradiative transition channels. To verify our speculation, the time-resolved PL spectra are presented in Fig. 1c. **BDP** showed a monoexponential decay at 730 nm with a value of 1.8 ns, while **2I-BDP** exhibited two exponential decay components at 720 nm with a value of 450 ps (60%, the amplitude for each component) and 1.7 ns (40%). The average lifetime of **2I-BDP** was estimated to be 0.95 ns (0.45 × 60% + 1.7 × 40%). Such a reduced fluorescence lifetime from 1.8 ns to 0.95 ns indicated the occurrence of additional nonradiative decay channels in **2I-BDP**, which is well consistent with the measurement of the PL quantum yield.

As is known, nonradiative transition mainly has two pathways, namely ISC and internal conversion. The former one is useful to produce ^1^O_2_ for PDT while the latter one generates heat for photothermal therapy.^18^ It is thus reasonable to conclude that these additional nonradiative decay channels in **2I-BDP** could lead to enhanced photothermal conversion or *Φ*_Δ_. Given the negligible photothermal conversion (Fig. S9†), **2I-BDP** is most likely to possess a very high *Φ*_Δ_. Therefore, characteristic ^1^O_2_ emission at 1270 nm was tested to validate whether **2I-BDP** has the capability to generate ^1^O_2_ (Fig. 1d).^19^ As compared with the negligible ^1^O_2_ emission in **BDP**, **2I-BDP** exhibited a strong emission at 1270 nm, suggesting an outstanding ^1^O_2_ generation. Furthermore, the calculated *Φ*_Δ_ of **2I-BDP** was up to 88% by using methylene blue (MB, *Φ*_Δ_ = 52% in methanol) as a standard and 1,3-diphenylisobenzofuran as a chemical trap (Fig. S10†).^20^ To our knowledge, this is the highest *Φ*_Δ_ among those of reported near-infrared PSs, which provides enormous potential for highly efficient PDT.

### ISC dynamics study

**Fig. 2:**
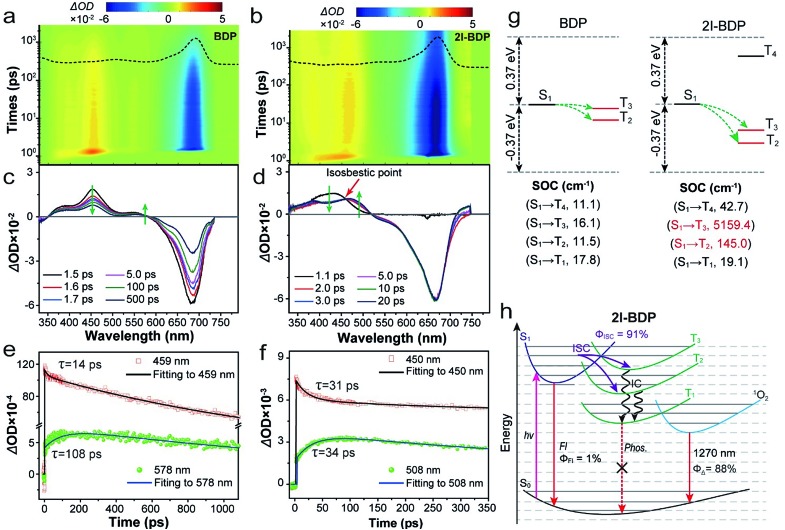
(a, b) 2D pseudocolor fs-TA spectra of **BDP** and **2I-BDP** in methanol (0.1 mg mL^–1^) following photoexcitation with a 650 nm laser pulse. The ground state absorption spectrum is presented in the top panel as dashed lines. (c, d) fs-TA spectra of **BDP** and **2I-BDP** at different pump-probe delay times. (e, f) Kinetic traces and fitting lines of **BDP** and **2I-BDP** taken through the representative singlet and triplet ESA peaks. (g) Schematic diagrams showing the computed energy levels and the probably occurring channels of ISC from the S_1_ state to its higher- or lower-lying triplet states (T_*n*_) possible. The triplet states marked in red contain the same transition components as S_1_. Average spin–orbit coupling (SOC) matrix elements between the respective singlet and triplet states (the larger the SOC value, the higher the possibility of ISC). (h) Schematic representation of ISC dynamics and photosensitization processes taking place in **2I-BDP.** Fl: fluorescence, Phos: phosphorescence, and IC: internal conversion.

To get the underlying reason for the ultrahigh *Φ*_Δ_ in **2I-BDP**, ultrafast excited-state dynamic behaviors in **BDP** and **2I-BDP** were systematically inspected by employing femtosecond transient absorption (fs-TA) spectroscopy. According to the steady-state absorption spectra (Fig. 2a), excitation with a 650 nm laser pulse has enough energy to populate **BDP** and **2I-BDP** ground states (S_0_) to the first excited state (S_1_). Both **BDP** and **2I-BDP** show a distinct negative absorption band ranging from 580 to 700 nm (Fig. 2a and b), which is quite consistent with their steady-state absorption spectrum and thus assigned to their ground state bleaching (GSB). For exploring detailed dynamic behaviors from the positive absorption band assigned to the excited-state absorption (ESA), fs-TA plots at different pump-probe delay times were extracted from Fig. 2a and b. As shown in Fig. 2c and d, a strong positive absorption band in the range of 360–580 nm for **BDP** and 340–515 nm for **2I-BDP** was clearly observed. Within the sub-10 ps domain (<10 ps, Fig. S11†), **BDP** exhibited two distinct fast rising positive absorption peaks at *ca.* 390 and 450 nm, while **2I-BDP** showed one fast rising positive absorption band from 340 to 525 nm. With the increase of delay time (Fig. 2c and d), the absorption intensity at *ca.* 390 and 450 nm for **BDP** and in the range of 390 to 460 nm for **2I-BDP** gradually decreases. Meanwhile, accompanied by the decay of the abovementioned peaks, a particularly noticeable peak at *ca.* 508 nm increased promptly in **2I-BDP**, while only an unnoticeable peak at *ca.* 578 nm increased very slowly in **BDP**. This phenomenon usually indicates the formation of new ESA species. Moreover, it is quite evident that these new emerging ESA species possess a longer lifetime than the decay ones.

In order to clarify the assignment of these ESA species, a singular value decomposition (SVD) method was applied to the kinetic traces at representative wavelengths (Fig. 2e and f). As shown in Fig. 2e, the kinetic decay from **BDP** at 459 nm revealed two exponential decay components with a value of 14 ps (7%) and 1603 ps (93%). This long-lived component corresponds well with the fluorescence lifetime (1.8 ns) of **BDP** obtained from time-resolved PL spectra (Fig. 1c). Therefore, we assigned the absorption band from **BDP** around 459 nm to the singlet ESA, which occurred within 14 ps. Considering that the triplet excited state has a longer lifetime than the singlet excited state, the new emerging ESA species from **BDP** that appeared around 578 nm could be assigned its triple ESA. The kinetic decay from **2I-BDP** at 450 nm in Fig. 2f also revealed two exponential decay components with a value of 31 ps (23%) and 18.7 ns (77%). This long lifetime component can be assigned to the triplet ESA. Moreover, an isosbestic point was observed at 460 nm (Fig. 2d), which was an indication of the conversion from the singlet state to the triplet state through ISC.^21^ On the basis of these data, we can infer that **2I-BDP** has a spectral overlap between the singlet and triplet ESA. The peak of **2I-BDP** around 508 nm could be assigned to its triplet ESA, and the peak of **2I-BDP** around 450 nm should be identified as the combination of singlet and triplet ESA. In addition, the rise time of the triplet ESA at 578 nm for **BDP** and 508 nm for **2I-BDP** was estimated to be 108 and 34 ps, respectively. The rate of ISC (*k*_ISC_) was determined to be 9.3 × 10^9^ s^–1^ for **BDP** and 2.9 × 10^10^ s^–1^ for **2I-BDP**, based on the rise time (=1/[*τ*_(T, rise)_]). Such a significantly larger *k*_ISC_ highlighted a faster ISC process in **2I-BDP**.

Furthermore, we performed first-principles time-dependent density functional theory (TD-DFT) investigations on the singlet and triplet excited states of **BDP** and **2I-BDP**. It is generally accepted that ISC occurred when two requirements were simultaneously satisfied: small singlet–triplet energy gap (Δ*E*_ST_) and same orbital transition component involved singlet and triplet states.^11^,^22^,^23^ Based on simple energetic arguments (Table S1 and S2†), there are 2 and 3 channels of T_*n*_ with small Δ*E*_ST_ (±0.37 eV) from *E*_S1_ in **BDP** and **2I-BDP**, respectively (Fig. 2g). Combining with the orbital transition components, the other factor needed for the occurring of ISC, only T_2_ and T_3_ contain the same orbital transition components as their respective S_1_ (Table S3 and S4†), Fig. 2g presents two facile ISC channels in **BDP** and **2I-BDP** (S_1_ → T_2_ and S_1_ → T_3_). Furthermore, to evaluate the ISC efficiency theoretically, we computed the average spin-orbital coupling (SOC) matrix elements as facile ISC could be distinguished by the high SOC values. The several orders of magnitude larger average SOC matrix elements in S_1_ → T_2_ and S_1_ → T_3_ channels (highlighted in red color in Fig. 2g) of **2I-BDP** theoretically supported its highly efficient ISC. Such a significant enhancement of the SOC value in **2I-BDP** should be ascribed to the iodine-induced heavy atom effect, in which the iodine atom promotes mixing of the singlet and triplet states of the excited chromophore. Finally, according to a reported method,^24^ the *Φ*_ISC_ = [1/[*τ*_(T, rise)_]]/[1/[*τ*_(S, decay)_]] was experimentally estimated to be 13% for **BDP** and 91% for **2I-BDP**, which is quite consistent with calculation and also supported the remarkably enhanced *Φ*_Δ_ in **2I-BDP**. To our knowledge, the *Φ*_ISC_ magnitude of **2I-BDP** is the highest in NIR-absorptive organic materials,^25^ providing enormous potential for the applications ranging from PDT, photocatalysis, and optoelectronic devices.

From the calculation and excited-state dynamics investigations, Fig. 2h schematically illustrates the ISC dynamics in **2I-BDP**. After photoexcitation, **2I-BDP** in the ground state (S_0_) was rapidly populated into S_1_. Then radiative (fluorescence) and nonradiative deactivation (IC and ISC) processes take place. Among the nonradiative deactivation processes, ISC takes place rapidly (within 34 ps) yet efficiently (*Φ*_ISC_ = 91%) through multiple channels to populate the triplet excited-state. We attribute this significantly enhanced *Φ*_ISC_ to the iodine-induced heavy-atom effect, which not only remarkably accelerates the ISC process but also enhances the ISC channel. Finally, energy in the higher triplet excited energy level transforms into the T_1_*via* IC, which substantially undergoes a photosensitization process to generate ^1^O_2_ for PDT.

### *In vitro* PDT experiments

**Fig. 3:**
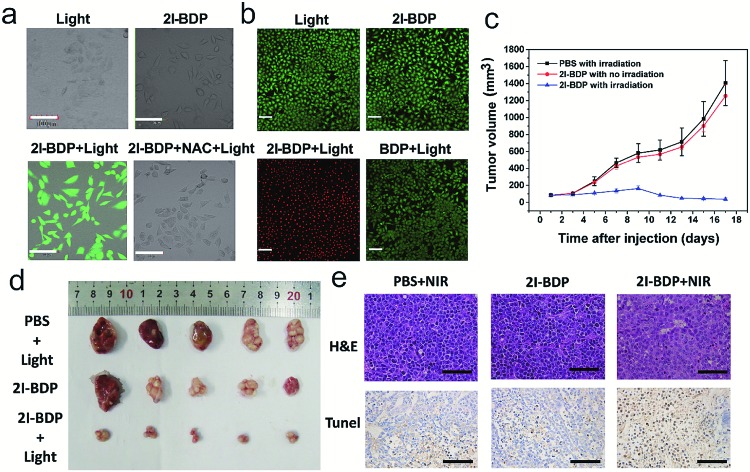
(a) Confocal fluorescence images of MCF-7 cells incubated with DCF-FA and **2I-BDP** under LED lamp irradiation. The scale bars are 100 μm. (b) Live/dead assay of MCF-7 cells. Green colour represents live cells, and red colour represents dead cells. The scale bars are 140 μm. (c) The tumor growth curves with different treatments. Error bars indicate SD (*n* = 6). (d) Gross solid tumor images of mice injected with different formulations 17 days post treatment. (e) The representative of the tumor sections examined by H&E staining and TUNEL. The scale bars are 100 μm.

In view of the key role of reactive oxygen species (ROS, *e.g.*, ^1^O_2_) in PDT, 2,7-dichlorofluorescein diacetate (DCFH-DA) that can react with ROS to form green fluorescent 2,7-dichlorofluorescein (DCF) was used to trace the ^1^O_2_ generation of **2I-BDP** in living cells.^26^ In MCF-7 cells treated with pure LED lamp irradiation or **2I-BDP**, there was no observable fluorescence (Fig. 3a). In contrast, a bright green fluorescence was observed in the presence of **2I-BDP** and LED lamp irradiation, indicative of ^1^O_2_ generation. Furthermore, *N*-acetylcysteine (NAC) as a singlet-oxygen scavenger was added to the MCF-7 cells with **2I-BDP** to uncover the influence of ^1^O_2_ in green fluorescence. The significantly inhibited green fluorescence highlighted the key role of ^1^O_2_ in green fluorescence. Taken together, these observations demonstrated that **2I-BDP** has the capability to generate ^1^O_2_ even in a complex biological environment.

With these outstanding properties, we could not wait to evaluate the PDT effect of **2I-BDP**. Initially, confocal fluorescence microscopy demonstrated that both **2I-BDP** and **BDP** could enter MCF-7 cells easily and distribute in the cytoplasm (Fig. S12†). Then, we studied the *in vitro* PDT efficacy of **2I-BDP** by employing calcein AM and propidium iodide (PI) cellular viability kits. The live/dead cells were differentiated by calcein AM (live cells, green fluorescence) and PI (dead cells, red fluorescence) co-staining after PDT treatment.^27^ In Fig. 3b, LED lamp irradiation or **2I-BDP** didn't induce obvious cell apoptosis, suggesting the negligible dark-cytotoxicity of **2I-BDP**. In sharp contrast, bright red fluorescence appeared in the cells treated with the cooperation of **2I-BDP** and LED lamp irradiation. Meanwhile, the morphology of cells showed significant changes (cell shrinkage and the formation of numerous blebs), revealing the excellent PDT effect of **2I-BDP**. To further evaluate the PDT effect of **2I-BDP** quantitatively, typical flow cytometry assay of MCF-7 cells was conducted. Upon the cooperation of **2I-BDP** and LED lamp irradiation, the late-stage apoptotic cancer cells increased to 90.1% (Fig. S13†), while there were no obvious changes in cell viability when the cells were treated with either irradiation or **2I-BDP** alone. From the typical 3-(4,5-dimethylthiazol-2-yl)-2,5-diphenyl-tetrazolium bromide (MTT) assay (Fig. S14†),^28^**2I-BDP** exhibited a negligible dark-cytotoxicity even at high concentration (100 μg mL^–1^) and an extremely high phototoxicity under the irradiation of the LED lamp at 660 nm (density of *ca.* 10 mW cm^–1^). In contrast, **BDP** showed no obvious dark-cytotoxicity and phototoxicity, which was consistent with its negligible *Φ*_Δ_. From these results, we can conclude that both **BDP** and **2I-BDP** have negligible cell dark-cytotoxicity but the phototoxicity of **2I-BDP** was so far superior to that of **BDP**.

### *In vivo* PDT therapy

Encouraged by the distinguishing PDT ability of **2I-BDP** toward cancer cells, *in vivo* therapeutic effects were then tested in MCF-7 tumor-bearing mice. For a better biocompatibility, **2I-BDP** was further encapsulated within a biodegradable copolymer DSPE-mPEG_5000_ to form water-soluble nanoparticles (Fig. S15†) through the nanoprecipitation method.^29^,^30^ In NIR fluorescence imaging (Fig. S16†), the fluorescence signal intensity at the tumor site reached a maximum after 4 h injection, which gave the optimal accumulation time point of **2I-BDP** for *in vivo* PDT. Such passive accumulation of **2I-BDP** in tumor sites should be ascribed to the enhanced permeability and retention (EPR) effect, because nanoparticles with size between 30 and 200 nm are believed to exhibit EPR-based tumor targeting.^31^ Meanwhile, *ex vivo* imaging of major organs harvested from those mice 4 h post injection further verified the tumor accumulation of **2I-BDP** (Fig. S17†).

After that, the *in vivo* PDT efficacy of **2I-BDP** was validated with a MCF-7 tumor-bearing mouse model. According to the NIR imaging results, the PDT treatment was conducted 4 h after intravenous injection because the accumulation reaches its maximum at this time point. After post injection of **2I-BDP** (100 μg mL^–1^, 150 μL) *via* the tail vein for 4 h, the tumor was exposed to 660 nm LED lamp irradiation with an ultralow power density of 10 mW cm^–2^ for 30 min. Without LED lamp irradiation, the tumor volume of **2I-BDP** injected mice increased as quickly as that of saline injected mice (Fig. 3c and d), indicating that pure **2I-BDP** or LED lamp irradiation was not able to inhibit tumor growth. In stark contrast, upon LED lamp irradiation, the tumor volume of **2I-BDP** injected mice was significantly inhibited, which is well consistent with the high phototoxicity of **2I-BDP** toward MCF-7 cells. Moreover, the body weight of all three groups remained stable, implying a negligible toxicity of all treatments (Fig. S18†). To further verify the PDT effect of **2I-BDP**, we applied the hematoxylin-eosin (H&E) and the terminal deoxynucleotidyl transferase dUTP nick end labeling (TUNEL) staining assay 17 days after treatment. Compared with the control groups treated with **2I-BDP** and LED lamp irradiation alone, H&E stained tumor tissues treated with **2I-BDP** and LED lamp irradiation exhibited a prominent necrosis and apoptosis of the tumor cells (Fig. 3e), confirming a successful destruction of the tumor cells. The TUNEL stained images displayed a higher level of cell apoptosis in the tumor tissue of the PDT group relative to that of control groups.^32^ These preliminary results demonstrated that **2I-BDP** can be used as a high-performance near-infrared PS for highly efficient PDT in living mice.

## Conclusions

In conclusion, we have deciphered the ISC dynamics in organic near-infrared BODIPY derivatives by experimental and theoretical investigations. Ultrafast fs-TA spectroscopy, in cooperation with calculation results, revealed a multi-channel yet exceptionally enhanced *Φ*_ISC_ (91%) in **2I-BDP**. Such an enhanced *Φ*_ISC_ endows **2I-BDP** with an ultrahigh *Φ*_Δ_ (88%), thus enabling a proof-of-concept application of highly efficient PDT *in vivo* under ultralow near-infrared light irradiation power density (10 mW cm^–2^). This work not only provides a novel organic near-infrared PS with ultrahigh *Φ*_Δ_ but also enriches the understanding of ISC dynamics in organic near-infrared materials, which may provide valuable guidance for designing novel organic theranostic materials for clinical cancer treatment.

## Ethical statement

All animal experiments were performed in accordance with the NIH guidelines for the care and use of laboratory animals (NIH Publication no. 85-23 Rev. 1985) and approved by the Jiangsu Administration of Experimental Animals. The nude mice (6 weeks of age) were subcutaneously injected with MCF-7 cells (1 × 10^6^) suspended in 50 μL PBS at the right armpit.

All MCF-7 tumor bearing nude mice were purchased from Nanjing OGpharmaceutical Life Science Co., Ltd. and used according to the guideline of the Laboratory Animal Center of Nanjing OGpharmaceutical Life Science Co., Ltd.

## Conflicts of interest

All authors declare no conflict of interest.

## Supplementary Material

Supplementary information
