# Supplementary material for: Deciphering the intersystem crossing in near-infrared BODIPY photosensitizers for highly efficient photodynamic therapy
Source: Chem Sci. 2019 Jan 22;10(10):3096–102. doi: 10.1039/c8sc04840a (PMC6429462; doi:10.1039/c8sc04840a)
Supplement: Supplementary file 1 [file SC-010-C8SC04840A-s001.pdf]

Electronic Supplementary Material (ESI) for Chemical Science.
This journal is © The Royal Society of Chemistry 2019

Supporting Information

Deciphering the Intersystem Crossing in Near-Infrared BODIPY Photosensitizer for Highly-Efficient Photodynamic Therapy

Xiaofei Miao,[a,‡] Wenbo Hu,[b,‡] Tingchao He,[c] Haojie Tao,[a] Qi Wang,[b] Runfeng Chen,[a] Lu Jin,[a] Hui Zhao,[a] Xiaomei Lu,[b] Quli Fan[a] and Wei Huang,[a,b,d]*

[a] Key Laboratory for Organic Electronics and Information Displays & Institute of Advanced Materials (IAM), Jiangsu National Synergetic Innovation Center for Advanced Materials (SICAM), Nanjing University of Posts & Telecommunications, Nanjing 210023, China.

[b] Key Laboratory of Flexible Electronics (KLOFE) & Institute of Advanced Materials (IAM), Jiangsu National Synergetic Innovation Center for Advanced Materials (SICAM), Nanjing Tech University (NanjingTech), Nanjing 211816, China.

[c] College of Physics and Energy, Shenzhen University, Shenzhen 518060, China.

[d] Shaanxi Institute of Flexible Electronics (SIFE), Northwestern Polytechnical University (NPU), 127 West Youyi Road, Xi'an 710072, China.

Materials: 4-Hydroxybenzaldehyde, 4'-Hydroxyacetophenone, diethylamine, ammonium acetate, Boron trifluoride diethyl etherate, *N*-iodosuccinimide, N,N-Dimethylformamide (99.8%, SuperDry), 3-diphenylisobenzofuran (DPBF), 2,7-dichlorofluorescein diacetate (DCFH-DA) were purchased from Sigma-Aldrich(Shanghai, China). Diethylene Glycol 2-Bromoethyl Methyl Ethern was purchased from TCI (Shanghai, China). Nitromethane were purchased from Alfa Aesar (Shanghai, China). Potassium hydroxide, anhydrous sodium sulfate, chloroform, acetic acid were obtained from Energy Chemical Reagent Co. Ltd. (Shanghai, China). All the above-mentioned chemical agents and solvents were used as received from commercial suppliers without further purification.

Instruments and Characteristic: Nuclear magnetic resonance (NMR) spectra were measured by using a Bruker Ultra Shield Plus 400 MHz. Mass spectra were obtained on a matrix-assisted laser desorption/ionization time of flight mass spectrometry MS (MALDI-TOF, Bruker AutoFlex III system). The steady-state near-infrared absorption spectra and photoluminescence spectra were collected on a SHIMADZU UV-3600 PLUS ultraviolet-visible-near-infrared (UV-Vis-NIR) spectrophotometer and Edinburgh FLSP920 fluorescence spectrophotometer, respectively. The absolute fluorescence quantum yield was measured using Edinburgh FLSP920 fluorescence spectrophotometer equipped an integrating sphere. The fluorescence decay spectra were measured using an Edinburgh FLSP920 fluorescence spectrophotometer. The decay lifetime was measured using Fianium WhiteLase. fs-TA was performed by Newport Transient Absorption Spectrometer, in which a Spectra-physics Tsunami Oscillator (80 MHz, 800 nm) was used as the seed for a Spectra-Physics Spitre Regenerative amplier (1 kHz, 4 mJ).

Synthetic route for target molecule

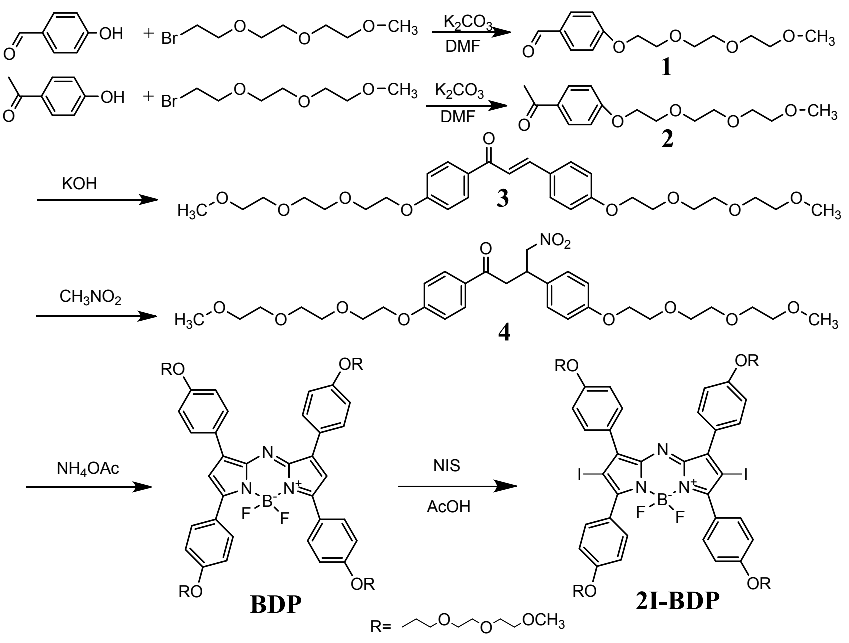

Fig.S1. Synthetic route of **BDP** and **2I-BDP**.

Synthesis of 1. 4-hydroxybenzaldehyde (1.74 g, 14.2 mmol) and (4.25 g, 30.8 mmol) K_2CO_3 were added to a 50 mL round-bottom flask. After removing the air by repeated vacuum-argon cycles, 10 mL of anhydrous DMF was injected, and the mixture was stirred at room temperature to make a homogeneous suspension. Then, a solution of diethylene glycol 2-bromoethyl methyl ether (3.24 g, 14.2 mmol) in 10 mL of anhydrous DMF was injected. The reaction mixture was stirred at 65 °C overnight. Then it was transferred to a separatory funnel and extracted with CH_2Cl_2 (×3). The organic phase was dried over anhydrous $MgSO_4$. The solvent was concentrated on a rotary evaporator for further purified by column chromatography on silica with ethyl ether as an eluent. The final product 1 was obtained as a yellowish oil. (3.98 g, yield: 80%). ^{1}H NMR (400 MHz, $CDCl_3$): δ = 9.85 (s, 1H), 7.85 – 7.77 (m, 2H), 7.06 – 6.93 (m, 2H), 4.25 – 4.15 (m, 2H), 3.90 – 3.84 (m, 2H), 3.72 (td, *J* = 3.9, 0.9 Hz, 2H), 3.69 – 3.59 (m, 4H), 3.52 (dd, *J* = 5.7, 3.6 Hz, 2H), 3.35 (s, 3H). ^{13}C NMR (101 MHz, $CDCl_3$): δ=190.88, 163.85, 131.96, 130.01, 114.88, 71.89, 70.88, 70.59, 69.45, 67.73, 59.04.

Synthesis of 2. 4-hydroxyacetophenone (1.93 g, 14.2 mmol) and (4.25 g, 30.8 mmol) K_2CO_3 were added to a 50 mL round-bottom flask. After removing the air by repeated

vacuum-argon cycles, 10 mL of anhydrous DMF was injected, and the mixture was stirred at room temperature to make a homogeneous suspension. Then, a solution of diethylene glycol 2-bromoethyl methyl ether (3.24 g, 14.2 mmol) in 10 mL of anhydrous DMF was injected. The reaction mixture was stirred at 65 °C overnight. Then it was transferred to a separatory funnel and extracted with CH_2Cl_2 (×3). The organic phase was dried over anhydrous $MgSO_4$. The solvent was concentrated on a rotary evaporator for further purified by column chromatography on silica with ethyl ether as an eluent. The final product 2 was obtained as a yellowish oil.(4.24 g, yield: 82%).^{1}H NMR (400 MHz, $CDCl_3$): δ =7.95 – 7.83 (m, 1H), 6.97 – 6.87 (m, 1H), 4.23 – 4.11 (m, 1H), 3.85 (dd, J = 5.4, 4.2 Hz, 1H), 3.72 (td, J = 3.9, 0.9 Hz, 1H), 3.69 – 3.60 (m, 2H), 3.55 – 3.50 (m, 1H), 3.35 (s, 1H). ^{13}C NMR (101 MHz, $CDCl_3$): δ =196.86, 162.71, 130.48, 114.26, 71.89, 70.86, 70.59, 69.51, 67.58, 26.36.

Synthesis of 3. Compound 2 (2.82 g, 10 mmol) was dissovled in ethanol (7 mL). Then the solution of compound 2 was gradually added to an aqueous solution of 10% KOH (30 mL) at 0 ºC. After stirring for 15 min, compound 1 (2.68 g, 10mmol) was added and stirred at 0 ºC for 15 min. The mixture was then allowed to attain room temperature and stirred overnight. Then the mixture was transferred to a separatory funnel and extracted with CH_2Cl_2 (×3). The organic phase was dried over anhydrous $MgSO_4$. The solvent was concentrated on a rotary evaporator for further purified by column chromatography on silica with eluent (AcOEt/MeOH 20/1). The final product 3 was obtained as colorless oil. (4.12 g, yield: 75%). ^{1}H NMR (400 MHz, $CDCl_3$): δ = 8.04 – 7.96 (m, 1H), 7.77 (t, J = 13.5 Hz, 1H), 7.57 (d, J = 8.8 Hz, 1H), 7.42 (d, J = 15.6 Hz, 1H), 7.04 – 6.87 (m, 2H), 4.25 – 4.13 (m, 2H), 3.87 (dd, J = 9.6, 5.5 Hz, 2H), 3.66 (ddd, J = 9.3, 5.1, 3.0 Hz, 4H), 3.54 (dd, J = 5.7, 3.6 Hz, 2H), 3.36 (s, 3H). ^{13}C NMR (101 MHz, $CDCl_3$): δ=188.77, 162.51, 160.73, 143.83, 131.44, 130.68, 130.10, 127.93, 119.58, 115.00, 114.38, 71.92, 70.88, 70.62, 69.59, 67.57, 59.06.

Synthesis of 4. A solution of compoud 3 (4 g, 7.5 mmol) in EtOH (10 mL) , KOH (20 mmol) and nitromethane (20 mmol) was added to a 100 ml two-neck flask and heated

at 60 ºC under reflux for 24 h. After cooling to room temperature, the mixture was transferred to a separatory funnel and extracted with CH_2Cl_2 (×3). The organic phase was dried over anhydrous $MgSO_4$. The solvent was concentrated on a rotary evaporator for further purified by column chromatography on silica with an eluent (AcOEt/PE 1/1). The final product 4 was obtained as a yellowish oil.(3.79 g, yield: 85%). ^{1}H NMR (400 MHz, $CDCl_3$): δ = 7.88 (d, J = 8.9 Hz, 1H), 7.17 (d, J = 8.6 Hz, 1H), 6.93 (d, J = 8.9 Hz, 1H), 6.86 (d, J = 8.7 Hz, 1H), 4.80 (dd, J = 12.3, 6.5 Hz, 1H), 4.62 (dd, J = 12.1, 7.9 Hz, 1H), 4.18 (dd, J = 9.1, 4.2 Hz, 1H), 4.11 – 4.06 (m, 1H), 3.90 – 3.85 (m, 1H), 3.85 – 3.80 (m, 1H), 3.73 (td, J = 5.9, 3.3 Hz, 2H), 3.66 (ddd, J = 9.2, 5.2, 2.8 Hz, 4H), 3.55 (dd, J = 5.7, 3.6 Hz, 2H), 3.37 (d, J = 3.9 Hz, 3H), 3.35 – 3.33 (m, 1H). ^{13}C NMR (101 MHz, $CDCl_3$): δ =195.46, 163.07, 158.28, 131.34, 130.32, 129.61,128.49,115.10,114.46,79.91,71.94,71.01,69.60,67.68,67.42,59.07,41.29,38.78.

Synthesis of BDP. A solution of compound 4 (1.1 g, 1 mmol) and ammonium acetate (1.5 g, 20 mmol) in ethanol (50 mL) was heated at 80 ºC under reflux for 48 h. After the solution was cooled to room temperature. The solvent was concentrated on a rotary evaporator for the next step. After removing the air by repeated vacuum-argon cycles, the crude intermediate (0.5 g, 0.44 mmol) was dissolved in the solution of Et_3N (0.45 g, 4.4 mmol). After stirring for 20 min at 0 ºC, $BF_3{\cdot}Et_2O$ (1.25 g, 8.8 mmol) was added and stirred at 0 ºC for 20 min. The mixture was then stirred at room temperature overnight. Ice water was added to the solvent to stop the reaction. After being stirred for one hour, cooling to room temperature, the mixture was transferred to a separatory funnel and extracted with CH_2Cl_2 (×3). The organic phase was dried over anhydrous $MgSO_4$. The solvent was concentrated on a rotary evaporator for further purified by column chromatography on silica with eluent (AcOEt/DCM 5/1). The final product **BDP** was obtained as a purple solid (0.15 g, yield: 30%). ^{1}H NMR (400 MHz, $CDCl_3$): δ = 8.07 – 7.99 (m, 8H), 7.00 (dd, J = 9.1, 2.4 Hz, 8H), 6.92 (s, 2H), 4.27 – 4.15 (m, 8H), 3.95 – 3.84 (m, 8H), 3.80-3.64(m,24H),3.57–3.54(m,8H),3.38(s,12H). ^{13}C NMR (101 MHz, $CDCl_3$) δ 160.95, 159.96, 157.78, 145.18, 142.73, 131.46, 130.70, 125.64, 124.52, 117.19, 114.76, 71.95, 70.90, 70.64, 69.66, 67.55, 59.05. MALDI-TOF-

MS(m/z): calcd for $C_{60}H_{78}BF_2N_3O_{16}$, exact mass: 1146.08; found: 1146.37.

Synthesis of 2I-BDP. Compound **BDP** (0.3 g, 0.26 mmol) and N-Iodosuccinimide (0.146 g, 0.65 mmol) were added to a 50 mL round-bottom flask. Then a solution of $CHCl_3$ and acetic acid (20 mL, 3:1) was injected and stirred at 30 ºC for 4 h. The solvent was concentrated on a rotary evaporator for further purified by column chromatography on silica with eluent (MeOH/$CHCl_3$, 1:9). The final product **2I-BDP** was obtained as a blue solid (0.1 g, yield: 32%). ^{1}H NMR (400 MHz, $CDCl_3$): δ = 7.79 (d, *J* = 8.9 Hz, 4H), 7.64 (d, *J* = 8.8 Hz, 4H), 7.01 – 6.93 (m, 8H), 4.23 – 4.15 (m, 8H), 3.93 – 3.85 (m, 8H), 3.76 (ddd, *J* = 6.4, 4.9, 2.3 Hz, 8H), 3.72 – 3.64 (m, 16H), 3.56 (dd, *J* = 5.7, 3.6 Hz, 8H), 3.38 (s, 12H). ^{13}C NMR (101 MHz, $CDCl_3$): δ=167.74 , 160.67 , 160.05 , 147.28, 144.83 , 132.41, *J* = 15.7, 130.86, 129.08, 124.82 , 123.72 , 114.03, 71.96, 70.89, 70.59, 68.18, 67.53, 59.02.MALDI-TOF MS (m/z): calcd for $C_{60}H_{76}BF_2I_2N_3O_{16}$, exact mass: 1397.34; found: 1397.87.

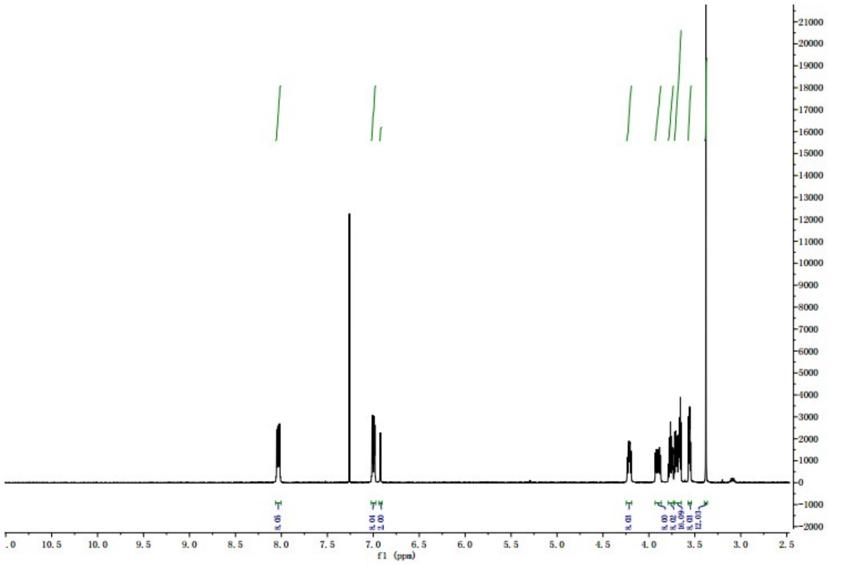

Fig.S2. ^{1}H NMR of **BDP**. (400 Hz, $CDCl_3$)

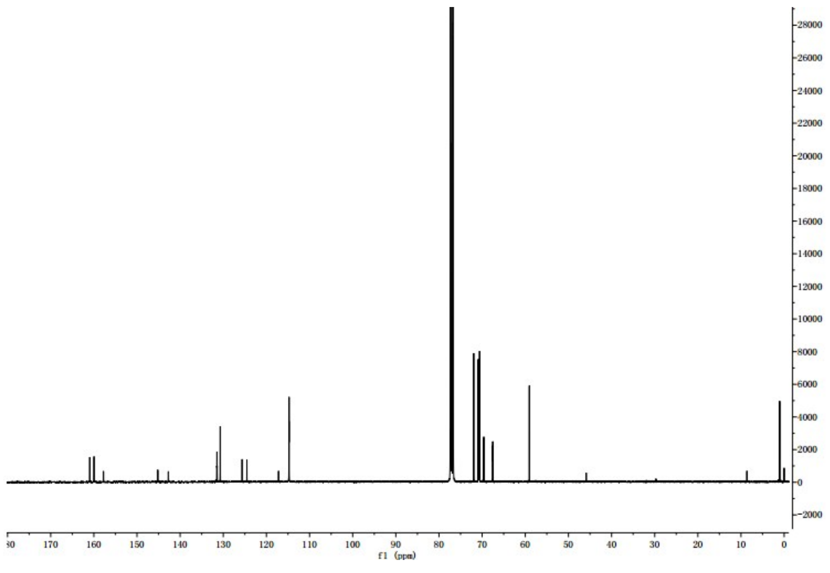

Fig.S3. ^{13}C NMR of **BDP**. (400 Hz, $CDCl_3$)

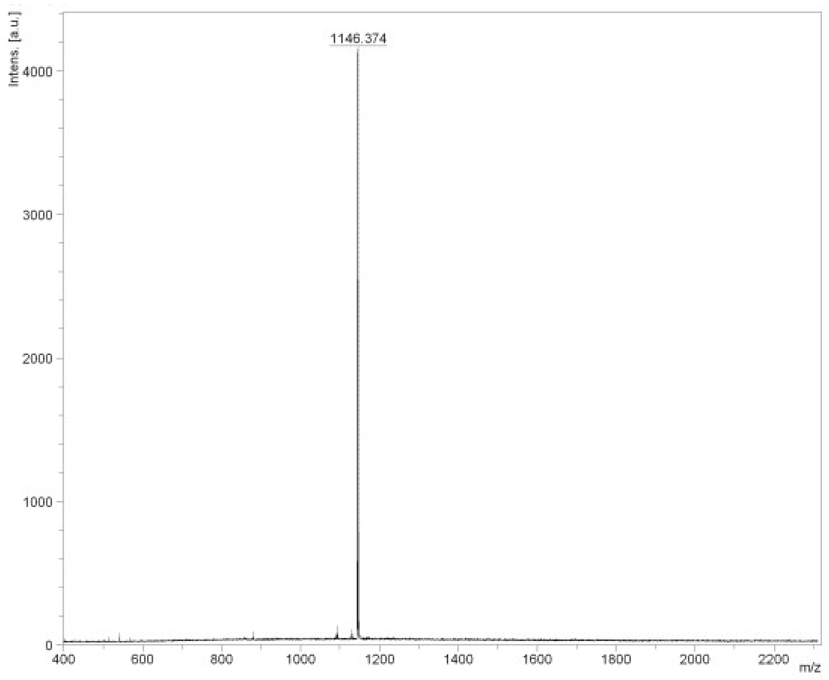

Fig.S4. HRMS chart of **BDP**.

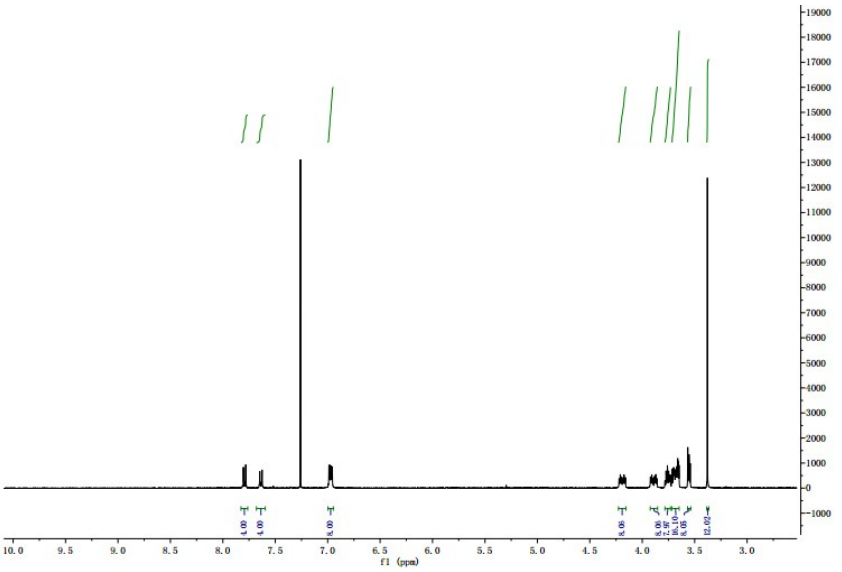

Fig.S5. ^{1}H NMR of **2I-BDP**. (400 Hz, $CDCl_3$)

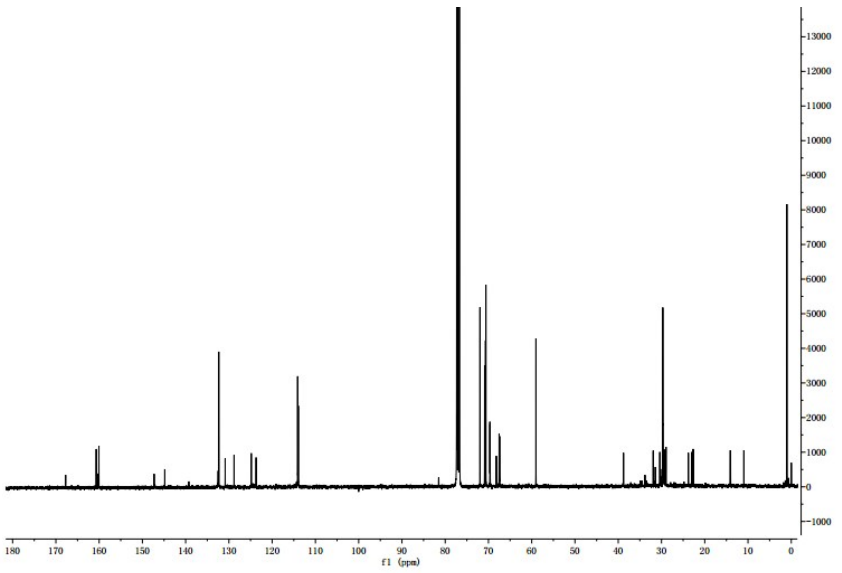

Fig.S6. ^{13}C NMR of **2I-BDP**. (400 Hz, $CDCl_3$)

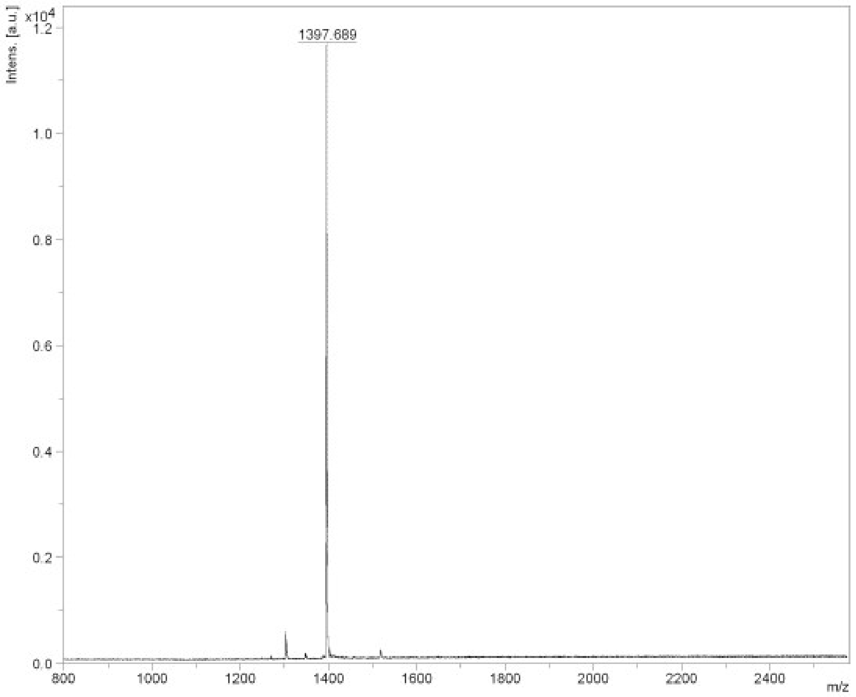

Fig.S7. HRMS chart of **2I-BDP**.

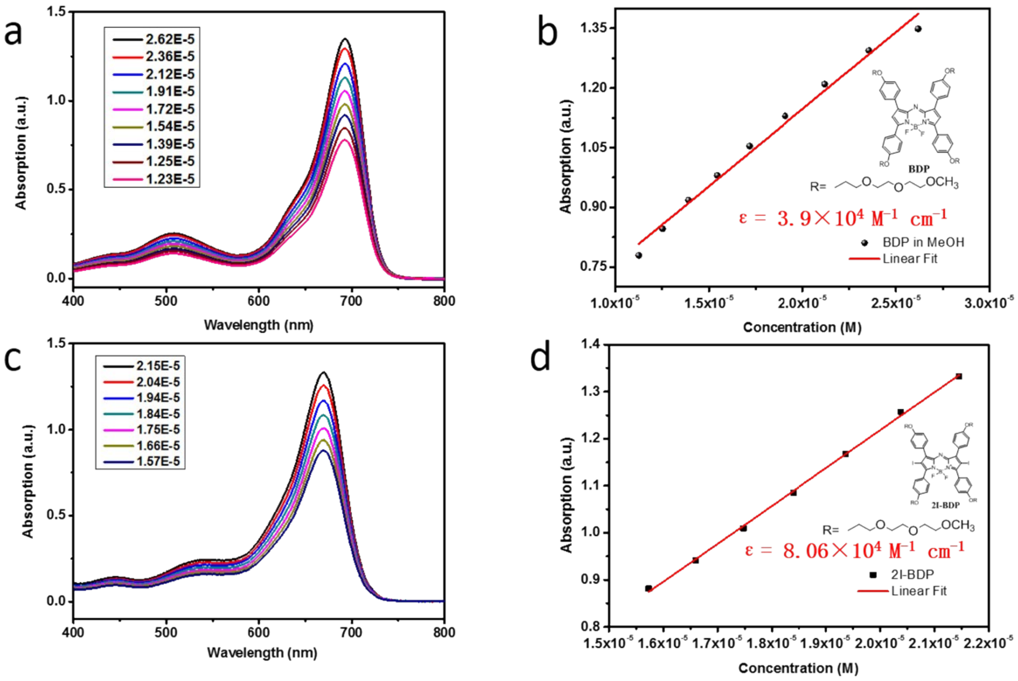

Fig.S8. (a) Concentration dependence of the UV-vis-NIR absorption of **BDP** in MeOH. (b) The plot of optical density at 690 nm versus concentration. (c) Concentration dependence of the UV-vis-NIR absorption of **2I-BDP** in MeOH. (d) The plot of optical density at 670 nm versus concentration. The straight line is a linear least-squares fit to the data.

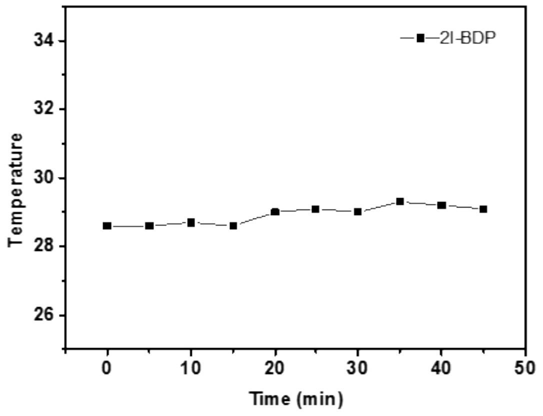

Fig.S9. Temperature curves of **2I-BDP** in MeOH with LED Lamp irradiation (660nm, 10 mW cm^{-1}).

Measurement singlet oxygen quantum yield (Φ_Δ): The singlet oxygen generation of **BDP** and **2I-BDP** was detected through monitoring oxidation of $_1O^2$ scavenger, 1, 3-diphenylisobenzofuran DPBF, with irradiation. The Φ_Δ quantum yields were calculated by using methylene blue in methanol with Φ_Δ= 0.52 as reference.[1] The absorbance of DPBF was adjusted to around 1.0 in methanol. Then the photosensitizer was added and its absorbance was adjusted to around 0.2–0.3. Then the cuvette was exposed to LED irradiation (660 nm) for 2 s, the irradiation intensity was fixed at 10 mW cm^{-2}. Then the absorbance of DPBF at 415 nm was monitored versus time. The singlet oxygen quantum yields (Φ_Δ) were calculated according to equation (1)

$\Phi_\Delta = \Phi_{(MB)} \times k_{(PS)} \times F_{(MB)} / k_{(MB)} \times F_{(PS)}$ ....(1)

k is the slope of a plot of difference in change in the absorbance of DPBF (415 nm) with irradiation time, and F is the absorption correction factor, which is given by $F = 1–10^{OD}$ (OD at the irradiation wavelength).

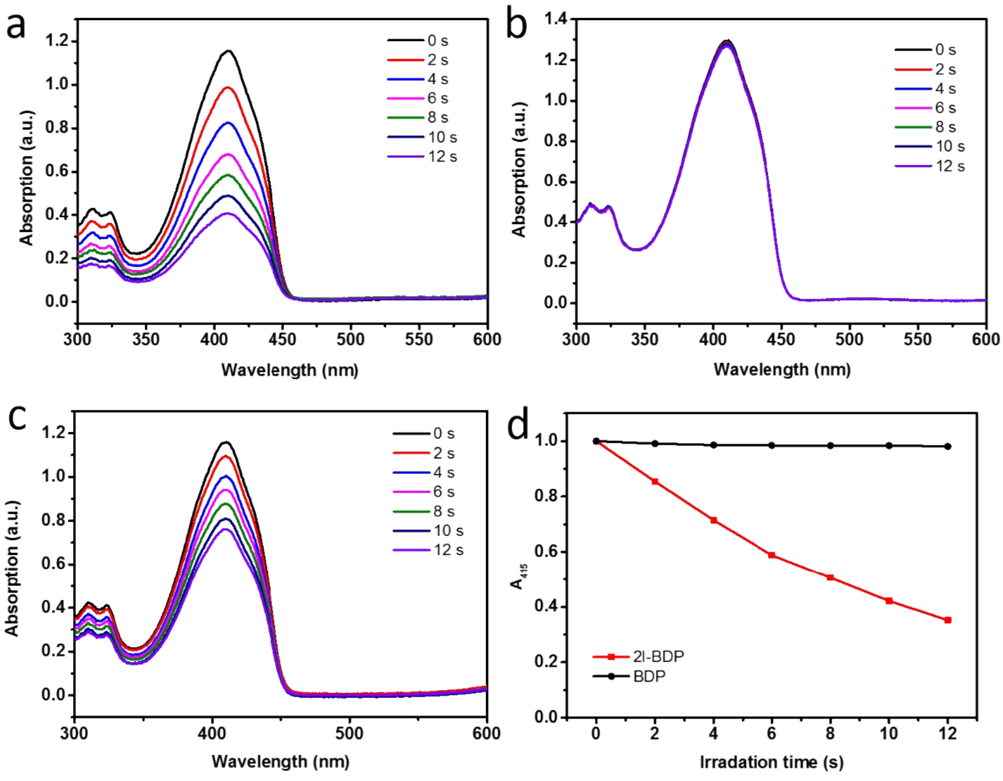

Fig.S10. (a) Change of DPBF UV-vis absorption spectra when **2I-BDP** was added as photosensitizer. (b) Change of DPBF UV-vis absorption spectra when **BDP** was added as photosensitizer. (c) Change of DPBF UV-vis absorption spectra when MB was added as photosensitizer. (d)Plots of change in consumption of DPBF at 415 nm versus the irradiation time of **BDP** and **2I-BDP**.

Femtosecond transient absorption (fs-TA) experiments: The measurements were performed using a pump-probe setup (18SI80466 Rev.1, Newport). The samples were pumped with the pulses (110 fs, 1000 Hz) at 650 nm, which were generated by a femtosecond mode-locked Ti–sapphire laser (Spectra Physics). The probe pulses (350–750 nm) were generated by focusing a small portion (~1.5 μJ) of the fundamental 800 nm laser pulses on a thin CaF2 plate. The linear polarization of the pump pulse was adjusted to be perpendicular to that of the probe pulse with a polarizer and a half-wave plate. The cross-polarization is intended to eliminate any contribution from coherent artifacts. Pump-induced changes of transmission (ΔT/T) of the probe beam were monitored using a monochromator/photomultiplier configuration with lock-in detection. The pump beam was chopped at 100 Hz, and this was used as the reference frequency for the lock-in amplifier.

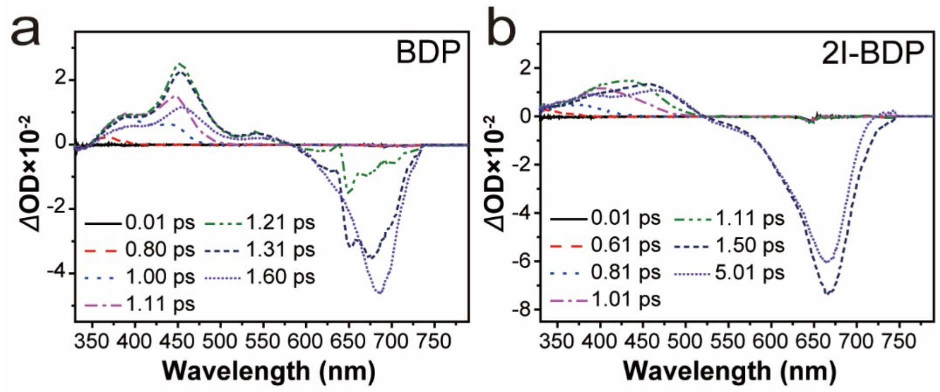

Fig.S11. fs-TA spectra of **BDP** and **2I-BDP** within sub-10 ps pump-probe delay times.

Time-dependent density functional theory (TD-DFT) calculations: The computations carried out by using Gaussian 09 program (Revision A. 02) and its time dependent formulation TD-DFT levels of theory. The ground (S_0) and lowest triplet (T_1) state geometries were fully optimized with the Becke's three-parameter exchange and correlation functional along with the Lee Yang Parr's correlation functional (B3LYP) using 6-31G (d) basis sets. In addition, the basis sets of iodine atom in **2I-BDP** was LanL2DZ. These optimized stationary points were further characterized by harmonic vibration frequency analysis to ensure that real local minima had been found. The excitation energies in the n-th singlet (S_n) and n-th triplet (T_n) states were obtained using the TD-DFT method based on an optimized molecular structure at ground state (S_0). The oscillator strength of **BDP** and **2I-BDP** were performed at the optimized ground (S_0) at the B3LYP level of theory using 6-31G (d) basis sets. In addition, the basis set of iodine atom in **2I-BDP** was LanL2DZ.

Spin-orbit coupling (SOC) matrix elements between the singlet excited states and triplet excited states were calculated with quadratic response function methods using the Dalton program. The SOCs of **BDP** and **2I-BDP** were performed at the optimized geometry of the lowest triplet excited state (T_1) at the B3LYP level of theory and cc-PVTZ basis set. In addition, the basis sets of iodine atom in **2I-BDP** was LanL2DZ.

Table S1. The singlet (S_n) states energy and oscillator strength of **BDP** and **2I-BDP**

	**BDP**	**2I-BDP**

	Energy (eV)	Oscillator Strength	Energy (eV)	Oscillator Strength
**S_1**	2.03	0.705	2.04	0.610
**S_2**	2.36	0.029	2.20	0.041
**S_3**	2.46	0.425	2.29	0.313
**S_4**	2.94	0.166	2.65	0.120
**S_5**	3.26	0.016	2.82	0.061
**S_6**	3.43	0.003	3.07	0.003
**S_7**	3.45	0.003	3.10	0.002
**S_8**	3.50	0.003	3.18	0.003
**S_9**	3.51	0.003	3.23	0.001
**S_{10}**	3.70	0.005	3.31	0.005

Tables S2. Theoretically calculated S_1-T_n energy gap and S_1/T_n spin-orbit coupling (SOC) constant of **BDP** and **2I-BDP**.

**S_1/T_n**	**S_1–T_n Energy Gap (eV)**		**SOC (cm^{-1})**	
	**BDP**	**2I-BDP**	**BDP**	**2I-BDP**
**S_1/T_1**	1.15	1.10	17.78	19.07
**S_1/T_2**	0.08	0.20	11.52	144.97
**S_1/T_3**	0.02	0.14	16.15	5159.38
**S_1/T_4**	-0.43	-0.25	11.08	42.68
**S_1/T_5**	-0.81	-0.46	64.18	10.23
**S_1/T_6**	-1.10	-0.82	6.36	25.93
**S_1/T_7**	-1.24	-0.90	3.24	8.59
**S_1/T_8**	-1.30	-0.96	4.04	16.97
**S_1/T_9**	-1.32	-0.98	10.55	22.04

**S_1/T_{10}**	-1.33	-1.03	19.63	4.26

Table S3. The singlet and triplet excited state transition configurations of **BDP** revealed by TD-DFT calculations. The matched excited states that contain the same orbital transition components of S_1 were highlighted in red.

	***n*-th**	**Energy (eV)**	**Transition configuration (%)**
**S_n**	1	2.03	H-1→L (2.69%)，H→ (98.75%)
**T_n**	1	0.88	H→L (61.08%)
	2	1.94	H-1→L (91.55%)，H-4→L (2.15%)，H-2→L (2.03%)
	3	2.01	H-10→L (2.40%)，H-3→L (2.21%)，H-2→L (90.88%)，H-1 →L (2.01%)
	4	2.46	H-4→L (92.09%)，H-2→L (2.28%)，
	5	2.83	H-4→L (88.58%)，H-1→L (2.28%)

Table S4. The singlet and triplet excited state transition configurations of **2I-BDP** revealed by TD-DFT calculations. The matched excited states that contain the same orbital transition components of S_1 were highlighted in red.

	***n*-th**	**Energy (eV)**	**Transition configuration (%)**
**S_n**	1	2.04	H-1→L (3.96%)、 H→L (94.89%)
**T_n**	1	0.88	H-10 →L (3.01%)、H-4 →L (2.19%) H →L (99.05%)
	2	1.84	H-4 →L (3.06%)、H-2→L (13.63%) H-1 →L (79.58%)
	3	1.91	H-3→L (2.13%)、H-2→L (80.06%) H-1→L (13.16%)
	4	2.29	H-3→L (93.57%)
	5	2.50	H-4→L (91.57%)、H-1→L (3.18%)

Cell culture: MCF-7 cells were cultured in DMEM culture medium supplemented with 10 % fetal bovine serum (FBS), 100 units mL^{-1} of penicillin and 100 g mL^{-1} of

streptomycin, and maintained in a humidified, 5 % carbon dioxide (CO_2) atmosphere at 37 °C. Then, cells were carefully harvested and split when they reached a certain confluence for following use.

Cellular uptake: The MCF-7 cells were seeded in a single dish plates for 24 h in a humidified, 5% carbon dioxide (CO_2) atmosphere at 37 °C. Then **BDP**/**2I-BDP** (50 μM) was added for 4 hours. After that, the cells were washed once with PBS buffer. Images of **BDP**/**2I-BDP** stained cells were visualized with a laser scanning confocal microscope.

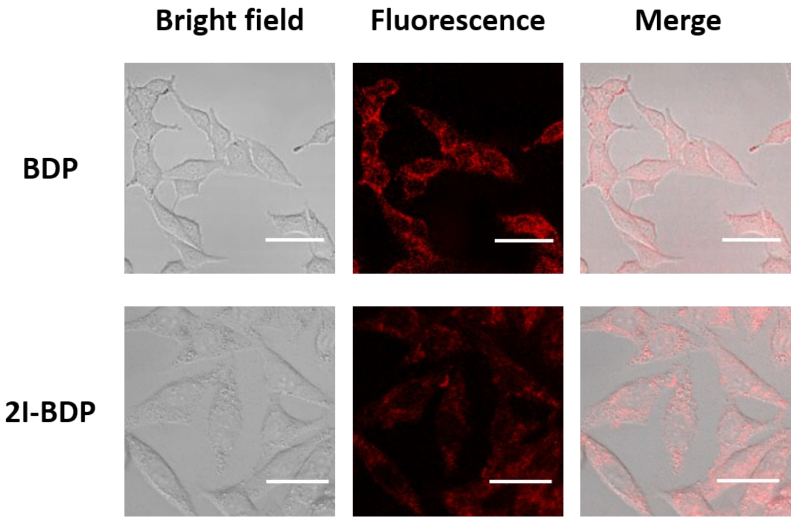

Fig.S12. Confocal laser scanning microscopic images of MCF-7 cells. The first line was **BDP** incubated for 24 h, scale bar represents 40 μm; the second line was **2I-BDP** incubated for 24 h, scale bar represents 30 μm. The first channel was bright field; the red channel was the fluorescence of **BDP** and **2I-BDP**; the last channel was overlapped.

ROS detection in live cells: MCF-7 cells were seeded in glass-bottom dishes and grown till 70 ~ 80% confluences. Subsequently, cells were incubated with **2I-BDP** (50 μM) for 6 h at 37 °C under 5% CO_2. Cells were then washed three times with PBS, after that incubated with DCFH-DA for 20 min. After LED lamp irradiation (660 nm, 10 mW cm^{-2}) for 20 min, the cells were imaged with a laser scanning confocal microscope. Fluorescence from DCFH-DA solution were recorded in 500-550 nm emission range with the excitation wavelength of 488 nm. In the presence of NAC, the irradiation-induced cell death was also examined.

Confocal Imaging of Photoinduced Cell Death: MCF-7 cells were seeded in the culture plates at 37 °C for 24 h, and then the cells were washed once with PBS. DMEM medium (2 mL) with 100 µg **BDP**/**2I-BDP** (50 µg/mL) was added. After the cells were further cultured for 6 h, the cells were stained with Calcein-AM and PI, the plates were irradiated under 660 nm light for 20 min (10 mW cm^{-2}), and the cell death imaging was visualized with a laser scanning confocal microscope. Under the excitation wavelength at 488 nm, collecting ranges were 500-550 nm for Calcein-AM emission and 600-650 nm for PI emission, respectively.

Flow Cytometry: MCF-7 cells were seeded in the six-well plates for 24 h, 37 °C. Then the cells were washed once with PBS. DMEM culture medium (2 mL) with 100 µg **BDP**/**2I-BDP** (50 µg/mL) was added for 6 h further incubation. The medium was then replaced with fresh culture medium and irradiated by 660 nm LED lamp at a power of 10 mW cm^{-2} for 20 min. Afterward, the cells were stained with Annexin V–FITC/PI according to the manufacturer's instruction, trypsinized, harvested, rinsed with PBS, resuspended, and subjected to perform flow cytometric assay using BD FACSCanto II flow cytometry.

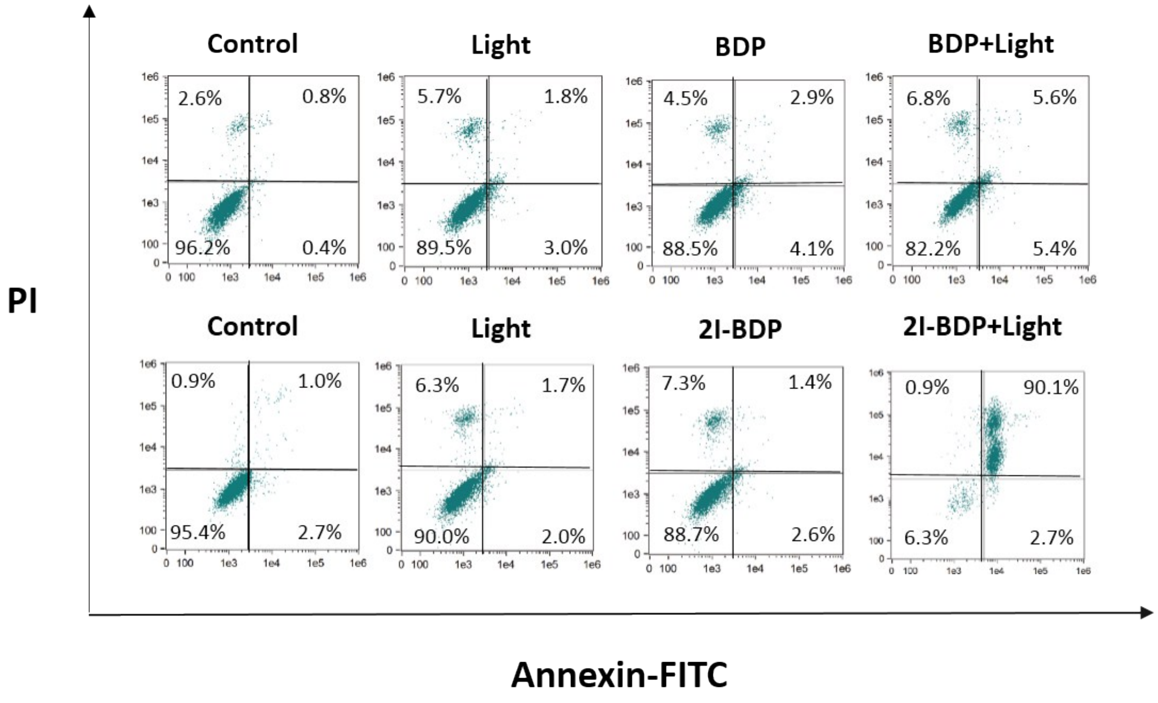

Fig.S13. Flow cytometry analyses of MCF-7 cell apoptosis induced by PDT.

Cellular cytotoxicity: To determine the cytotoxicity of **BDP** and **2I-BDP**, MCF-7 cells were used to carry out MTT assays. MCF-7 cells were seeded into four 96-well and plated in appropriate culture condition for 24 h. Then, **BDP/2I-BDP** were added at different concentrations (0, 5, 10, 20, 50, 100 μM) and MCF-7 cells were incubation for 12 h. Then two plates containing photosensitizers were irradiated with LED irradiation (660 nm, 10 mW cm^{-2}) for 20 min while the others were kept in dark. After illumination, these 96-well plates were incubated for additional 6 h at 37 °C.Finally, 20 μL MTT solution (5 mg mL^{-1}) were add to each well and cultivate for another 4 h at 37 °C. After that, we remove supematant and add 200 μL dimethyl sulfoxide to each well. The absorbance intensity was recorded by a PowerWave XS/XS2 microplate spectrophotometer at 490 nm. The cellular viability relative to the untreated cells (control group) was calculated following equation:

$$\text{Cell viability (\%)} = \frac{A_{\text{sample}}}{A_{\text{control}}} \text{x100\%}$$

in which A_{sample} and A_{control}, respectively, represent the average absorption of groups containing photosensitizers and untreated cells.

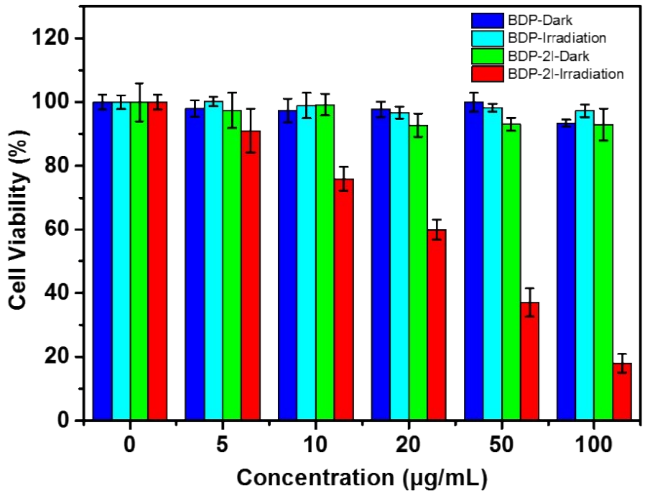

Fig.S14. MTT assay of MCF-7 cells viability of different concentrations of **BDP** and **2I-BDP**: **BDP**+ dark (blue), **BDP** + NIR (cyan), **2I-BDP** + dark (green), and **BDP** + NIR (red) after treatment.

Preparation of Water-Soluble 2I-BDP NPs: 2I-BDP (2.0 mg) in 2 mL THF was swiftly dropped into the DSPE-mPEG5000 aqueous solution (5.0 mg in 10 mL H_2O) under sonication. THF was then removed by argon blowing over the solution surface under stirring at 40 °C. A blue aqueous solution was then obtained. The aqueous solution was concentrated with a centrifugal filter and washed several times. The resultant products were concentrated and filtered through a 0.22 μm filter for the future experiments.

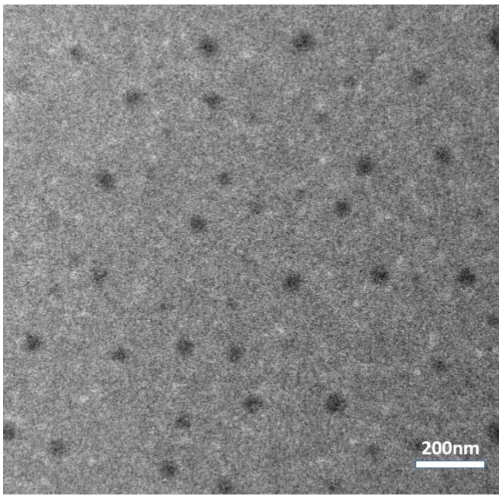

Fig.S15. TEM image of **2I-BDP** NPs, scale bar represents 200 nm.

Animals and tumor model: All MCF-7 tumor bearing nude mice were purchased from Nanjing OGpharmaceutical Life Science Co., Ltd. and used according to the guideline of the Laboratory Animal Center of Nanjing OGpharmaceutical Life Science Co., Ltd. The nude mice (6 weeks of age) were subcutaneously injected with MCF-7 cells (1×10^6 cells/50 μL) at the right armpit. The tumor volume of MCF-7 tumor-bearing mice was calculated as volume = $(a \times b^2)/2$ (a: length; b: width). After the tumor volumes were about 100 mm^3, mice were used for *in vivo* imaging and phototherapy.

In Vivo Fluorescence Imaging: MCF-7 tumor-bearing nude mice was injected with **2I-BDP** NPs (100 μg/mL, 150 μL) through tail intravenous injection. *In vivo* fluorescence imaging can be visualized on IVIS Lumina K (Perkin Elmer) at 720 nm.

In Vivo Antitumor Efficacy: The mice were randomly divided into 3 group (n = 6) and intravenous injected with (1) PBS with 660 nm LED lamp irradiation (150 μL), (2) **2I-BDP**(100 μg/mL, 150 μL), (3) **2I-BDP** with 660 nm LED lamp irradiation(100 μg/mL, 150 μL), respectively. The 660 nm LED lamp (10 mW cm^{-2}) was used to irradiate the tumor after 4 h injection for 30 min. The body weight and tumor volume were monitored every 2 d for 17 d. Then all mice were sacrificed, and the major organs (heart, liver, spleen, lung, kidney) and tumor were harvested and examined by H&E staining and TUNEL staining assay.

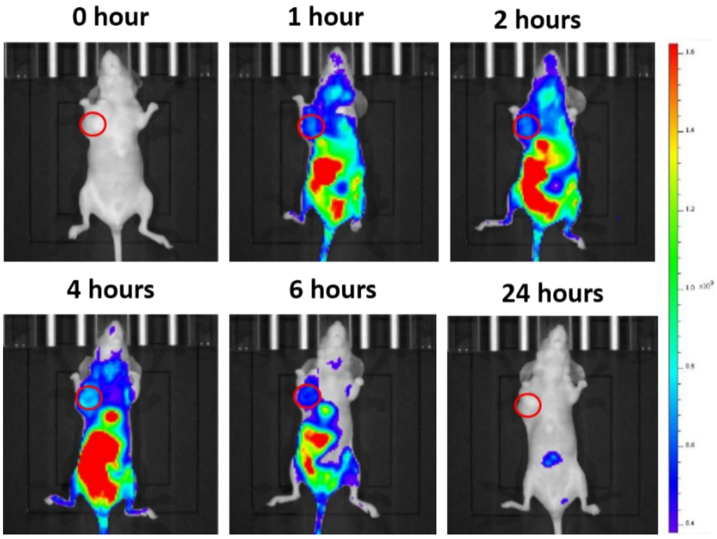

Fig.S16. Time-dependent *in vivo* NIR fluorescence images of MCF-7 tumor-bearing mice after intravenous injection of 100 μg mL^{-1}, 150 μL.

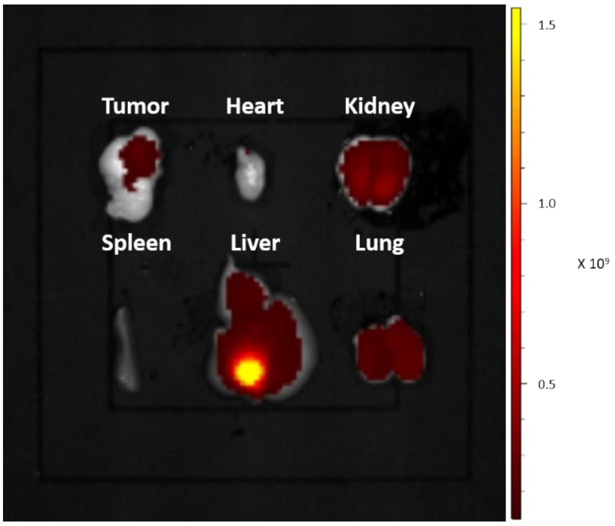

Fig.S17. *In vivo* distributions of **2I-BDP** (100 μg mL^{-1}, 150 μL). Time dependent *ex vivo* NIR fluorescence in major mice organs (heart, liver, spleen, lung, kidney, tumor after injection **2I-BDP** 4 h.

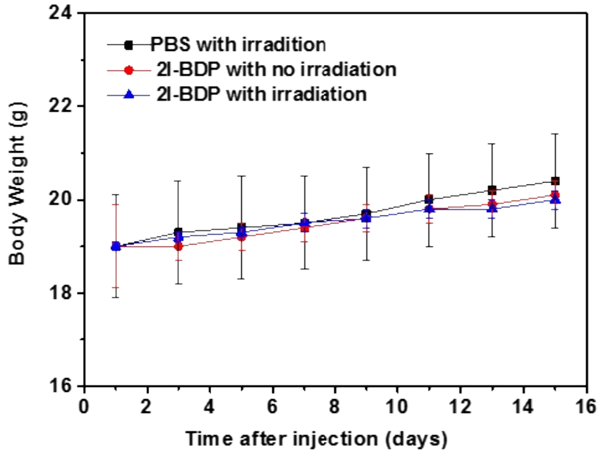

Fig.S18. Body weight changes with time of mice under different treatments.

Refer

(1) N. Adarsh, R. R. Avirah and D. Ramaiah, *Org. Lett.*, 2010, **12**, 5720.
